# Exposure to ambient particulate matter and biomass burning during pregnancy: associations with birth weight in Thailand

**DOI:** 10.1038/s41370-021-00295-8

**Published:** 2021-02-18

**Authors:** William Mueller, Kraichat Tantrakarnapa, Helinor Jane Johnston, Miranda Loh, Susanne Steinle, Sotiris Vardoulakis, John W. Cherrie

**Affiliations:** 1 Institute of Occupational Medicine, Edinburgh, UK; 2 Department of Social and Environmental Medicine, Faculty of Tropical Medicine, Mahidol University, Bangkok, Thailand; 3 School of Engineering and Physical Sciences, Institute of Biological Chemistry, Biophysics and Bioengineering, Heriot Watt University, Edinburgh, UK; 4 National Centre for Epidemiology and Population Health, Research School of Population Health, Australian National University, Canberra, ACT Australia

## Abstract

**Background:**

There is a growing evidence that exposure to ambient particulate air pollution during pregnancy is associated with adverse birth outcomes, including reduced birth weight (BW). The objective of this study was to quantify associations between BW and exposure to particulate matter (PM) and biomass burning during pregnancy in Thailand.

**Methods:**

We collected hourly ambient air pollutant data from ground-based monitors (PM with diameter of <10 µm [PM_10_], Ozone [O_3_], and nitrogen dioxide [NO_2_]), biomass burning from satellite remote sensing data, and individual birth weight data during 2015–2018. We performed a semi-ecological analysis to evaluate the association between mean trimester exposure to air pollutants and biomass burning with BW and low-birth weight (LBW) (<2500 g), adjusting for gestation age, sex, previous pregnancies, mother’s age, heat index, season, year, gaseous pollutant concentrations, and province. We examined potential effect modification of PM_10_ and biomass burning exposures by sex.

**Results:**

There were 83,931 eligible births with a mean pregnancy PM_10_ exposure of 39.7 µg/m^3^ (standard deviation [SD] = 7.7). The entire pregnancy exposure was associated with reduced BW both for PM_10_ (−6.81 g per 10 µg/m^3^ increase in PM_10_ [95% CI = −12.52 to −1.10]) and biomass burning (−6.34 g per 1 SD increase in fires/km^2^ [95% CI = −11.35 to −1.34]) only after adjustment for NO_2_. In contrast with these findings, a reduced odds ratio (OR) of LBW was associated with PM_10_ exposure only in trimesters one and two, with no relationship across the entire pregnancy period. Associations with biomass burning were limited to increased ORs of LBW with exposure in trimester three, but only for male births.

**Conclusion:**

Based on our results, we encourage further investigation of air pollution, biomass burning and BW in Thailand and other low-income and middle-income countries.

## Introduction

There is a growing evidence that exposure to ambient particulate air pollution during pregnancy is associated with adverse birth outcomes, including preterm birth [1], small for gestational age [2], and reduced birth weight (BW) [3, 4]. Determining the causality of these relationships is critical, since any adverse associations at birth may have life-long implications for health [5].

Steinle et al. [6] identified available systematic reviews and meta-analyses to evaluate the plausibility of a concentration-response function between ambient particulate matter (PM) and BW and low birth weight (LBW) (i.e., <2500 g [7]). Although the identified meta-analyses found large heterogeneity between individual studies, there emerged adverse associations between continuous and binary BW measures and exposures to PM with aerodynamic diameters of <10 μm (PM_10_) and <2.5 μm (PM_2.5_); for example, odds ratios (ORs) for LBW ranged from 1.01 (95% CI = 0.96–1.08) [8] to 1.05 (95% CI = 1.02–1.07) [9] per 10 µg/m^3^ of PM_10_.

To support and possibly explain these epidemiological findings, evidence from in vivo studies has shown that ambient PM, ultrafine carbon black, and diesel exhaust particles can cause reproductive and developmental toxicity following pulmonary exposure (reviewed by [10, 11]). The exact mechanism(s) underlying PM toxicity to the developing embryo/foetus is yet to be elucidated, but could involve particle translocation across the placenta, the ability of particles to cause altered placental function (e.g., due to the activation of inflammation and oxidative stress), and/or the presence of circulating inflammatory mediators produced in the mother following exposure of the lungs to PM (reviewed by [12]). In addition to PM, other ambient air pollutants, such as ozone (O_3_) and nitrogen dioxide (NO_2_), have been associated with reduced BW, possibly acting via similar mechanisms of oxidative stress and inflammation [13, 14].

The majority of the published epidemiological studies that investigate air pollution and birth outcomes originate from North America, Europe, and other higher income regions. In the review by Steinle et al. [6], only about 8% of the contributing studies in the meta-analyses were from Asia. Furthermore, these previous Asian studies have predominantly taken place in temperate regions (e.g., China, South Korea); BW in tropical nations may be additionally adversely affected by malaria and the presence of other infections or parasites occurring during pregnancy [15]. Also, as far as we are aware, none of the Asian studies were undertaken in settings where the population is routinely exposed to PM emissions from extensive burning of crop residues or forest, which can represent a significant PM source during these periods [16, 17]. The few existing studies of BW and maternal exposure to biomass burning have produced mixed results [18]. These knowledge gaps represent major limitations in the existing evidence base, particularly as large populations in Asia are exposed to high levels of PM from urban sources and biomass burning.

Our aim was to investigate in Thailand the relationship between continuous BW and LBW and cumulative exposure to PM_10_ air pollution and biomass burning during pregnancy. To help identify any sensitive windows, we assessed these exposures in the presence of gaseous air pollutants for the entire gestation length and also during potential critical periods, thus allowing for variation in pollution concentrations and sources occurring throughout the year. This study is part of the larger research project to study the effects of air pollution in Thailand: Thailand Air Pollution Health Impact Assessment.

## Methods

### Study setting

Thailand is situated in the tropics, with distinct wet (May–October), hot (March–May), and dry (November–February) seasons; with the exception of the north, temperatures are in excess of 30 °C for most of the year [19]. It is a fast developing upper middle-income country with a population of almost 70 million people. Thailand covers a land area of 513,000 km^2^, with around 41% for agriculture and 37% for forest [20]. Despite ongoing efforts by the government to reduce agricultural burning of crop residues, it is still widespread in Thailand: during 2019, there were ~35,000 fires detected [21]. Most of the fires in Thailand occur between December and April each year, and as a consequence, ambient particulate air pollution concentrations are significantly elevated during those months [22]. In addition to biomass burning, emissions from industry, traffic, and power plants are the main contributors of PM, carbon monoxide (CO) and nitrogen oxides (NO_*x*_), which can be precursors for the formation of O_3_ [23].

More than 99% of births are registered in Thailand [24]. Thailand is committed to providing universal healthcare for the population, funded from general taxation; as part of this provision, all births are attended by an experienced healthcare professional and 90.8% of expectant mothers attend four or more visits to antenatal care [25, 26].

### Birth outcome data

We obtained anonymised data on individual births during the years 2010–2018 for all Thailand provinces, except Bangkok (see below), from the Ministry of Public Health. The following variables were included in this dataset: infant sex, gravidity (i.e., number of previous pregnancies), BW (g), gestational age (in weeks), maternal age, date of birth, a binary indicator for any congenital anomalies, and International Classification of Diseases (ICD) coding for birth outcomes; however, the latter variable was mostly missing (>90%) and was therefore excluded. Some variables contained a high proportion (>50%) of missing data in the years 2010–2014, and total birth counts were lower in 2013 and 2014 compared to that of the other years with no apparent explanation. Therefore, based on the higher quality of the birth data in the most recent years of the dataset, we defined the study period to be 1 January 2015 to 30 April 2018.

We also collected for the years 2014–2018 anonymised individual birth data for Bangkok from the Bangkok Metropolitan Administration. This dataset contained variables for date of birth, sex of the baby, BW (g), and ICD coding to signify information such as single/multiple births and any pregnancy complications; however, gravidity, maternal age, and gestation age in weeks were not included in this dataset. Due to the omission of these important risk factors for BW [27], particularly given the typically more modest magnitude of risks associated with air pollution, we focussed our analysis on the Thai provinces with more complete data and thus excluded the Bangkok data from analysis.

### Exposure data

#### Air pollutants

The Thai Pollution Control Department (PCD) manages a network of ground-based stations to monitor air pollutants throughout the country. We collected hourly air quality data from the PCD network to identify all monitors during the study period with sufficient PM_10_, PM_2.5_, NO_2_, and O_3_ data. Mean daily data from a given monitor were considered to be sufficient if ≥75% of measurements (i.e., ≥18 h) of each pollutant were available on a given day [28] and ≥75% of such days were available during the study period. We selected as the main exposure PM_10_ concentrations instead of PM_2.5_, since only three monitors included sufficient PM_2.5_ monitoring data (compared to 13 monitors with PM_10_ data). The mean daily temperature (°C) and relative humidity (%) data, which were included with the air pollutant data from each station, were calculated in the same manner. For O_3_, maximum daily values of the 8-h rolling average were calculated, where six or more hours of data were available [29].

Each province was assigned the mean daily value from monitors with sufficient data; the average value was calculated if data were available from multiple monitors on the same day. These daily data were used to generate average trimester exposures over three 90-day periods preceding the date of birth, where at least 68 days (i.e., ≥75%) with data were available in each trimester. Exposure over the entire pregnancy was calculated as the mean of the three trimesters. Only those monitors with sufficient data (i.e., <25% missing data) for all pollutants (i.e., PM_10_, NO_2_, O_3_) were eligible for analysis (*n* = 12; Table S1).

#### Biomass burning

As an indicator for biomass burning, we obtained satellite remote sensing data from NASA’s Visible Infra-red Imaging Radiometer Suite sensor on the daily number of fires in Thailand during the study period at a spatial resolution of 375 m [30]. We excluded any fires demarcated as low confidence and retained only those categorised as vegetation fires. To standardise the data, we summed the number of fires in each province for each trimester and the entire pregnancy period, as described above, and divided by the area of the province; these values were then converted to z-scores for analysis.

### Statistical analysis

**Fig. 1:**
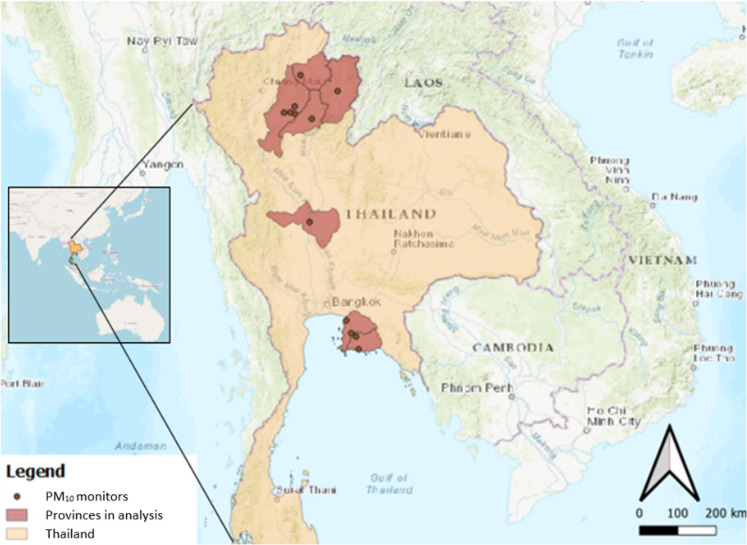
Map of the study area in Thailand, showing the included provinces (shaded) and locations of air pollution ground monitors.

We used a semi-ecological study design to develop linear and logistic regression models to assess, respectively, the association between exposure to province-level averages of particulate air pollution and biomass burning during pregnancy with two health outcomes from individual birth data: a continuous measure (g) and a binary outcome for LBW (defined as <2500 g). We included in the models covariates which have been previously shown to be important predictors of BW and for which we had data: gestation age in weeks, sex [31], gravida (continuous), and mother’s age (cubic spline with three knots based on lower BIC values) [27]. In Thailand, PM levels demonstrate strong seasonal trends; instead of adjusting for season, but to incorporate seasonal differences that might impact birth outcomes [32], we calculated an average heat index (HI) for each trimester based on temperature and relative humidity using Steadman’s formula, as presented in Anderson et al. [33]. We also accounted for potential unmeasured regional differences and long-term trends in BW by including covariates for province and year, respectively; this would help model differences in BW according to the timing, and thus exposure, of gestation. Only normal term pregnancies were included (i.e., gestation age of 37–41 weeks) with a maternal age of 14–60 years and a maximum of 20 previous pregnancies. Analysis was restricted to BWs of 1000–5200 g (i.e., ±5 standard deviations above and below the mean [34]). Analysis was conducted using those births with complete data in those provinces with <25% missing air pollution data (*n* = 7; Fig. 1).

Three classes of models are presented for each outcome: (1) model 1 unadjusted, (2) model 1 + all above covariates, (3) model 2 + other individual air pollutants. Regression coefficients were rescaled for all pollutants to represent changes per 10 µg/m^3^; O_3_ and NO_2_ were converted to µg/m^3^ from ppb using conversion factors of 1.96 and 1.88, respectively [35]. We adjusted for gaseous air pollutants in the models of PM_10_ and biomass burning if spearman rank correlations were <0.7; thus we included NO_2_ and excluded O_3_. Regression models were run with either exposure in all three trimesters [36] or an average of the entire pregnancy. Residuals were examined using histograms and Q–Q plots. Additional analyses were run to examine effect modification in PM_10_ and biomass burning exposures with sex. To assess the robustness of the BW data, we used tests of proportion to determine if the last digit (or two) of BWs were more likely to be rounded to the nearest 10 and 100 g (i.e., different from 10% or 1% of all births). We used QGIS (v3.10.1-A Coruña) for geospatial analysis with biomass burning data and performed statistical analysis using Stata (v15.1).

## Results

During the study period (2015–2018), there were 83,931 eligible births (out of a total of 158,457; 53.0%) with complete exposure and birth data in the study area. The mean BW was 3112 g (SD = 408.5), with 5.3% of births categorised as LBW. Nearly one third (32.7%) of the births occurred in the 38th week, and just under half (48.4%) of babies born were female. The average mother’s age was 26.9 years, with an average of about two (1.9) previous pregnancies (Table 1). There was strong evidence of rounded BWs: 77.7% (vs 10.0% expected) of recorded weights had ‘0’ as the last digit and 10.3% (vs 1.0% expected) as the last two digits (*p* < 0.001 in each instance).

**Table 1:** Descriptive characteristics of the study sample (*N* = 83,931).

Characteristics	Mean (SD) or *n* (%)
Birth weight (g)	3112 (408.5)
Low birth weight (<2500 g)	
Yes	4413 (5.3%)
No	79,518 (94.7%)
PM_10_ (µg/m^3^)	
Trimester 1	40.2 (18.5)
Trimester 2	38.8 (17.2)
Trimester 3	40.2 (17.3)
Entire pregnancy	39.7 (7.7)
O_3_ (µg/m^3^)	
Trimester 1	78.9 (26.6)
Trimester 2	76.8 (23.9)
Trimester 3	78.7 (24.6)
Entire pregnancy	78.1 (12.8)
NO_2_ (µg/m^3^)	
Trimester 1	16.4 (7.0)
Trimester 2	16.4 (7.2)
Trimester 3	16.8 (7.7)
Entire pregnancy	16.5 (5.8)
Biomass burning (fires/km^2^)	
Trimester 1	0.06 (0.11)
Trimester 2	0.05 (0.09)
Trimester 3	0.05 (0.09)
Entire pregnancy	0.15 (0.15)
Sex	
Male	43,343 (51.6%)
Female	40,588 (48.4%)
Gestation	
37 weeks	11,496 (13.7%)
38 weeks	27,425 (32.7%)
39 weeks	24,009 (28.6%)
40 weeks	17,560 (20.9%)
41 weeks	3441 (4.1%)
Maternal age (years)	26.9 (6.5)
Gravidity	1.9 (1.0)
Heat index	
Trimester 1	31.1 (3.5)
Trimester 2	30.6 (3.3)
Trimester 3	30.2 (3.3)
Entire pregnancy	30.6 (2.5)
Year	
2015	14,865 (17.7%)
2016	23,856 (28.4%)
2017	37,161 (44.3%)
2018	8049 (9.6%)
Province	
Chon Buri	17,500 (20.9%)
Rayong	26,149 (31.2%)
Lampang	14,186 (16.9%)
Phrae	1684 (2.0%)
Nan	6265 (7.5%)
Phayao	5713 (6.8%)
Nakhon Sawan	12,434 (14.8%)

**Fig. 2:**
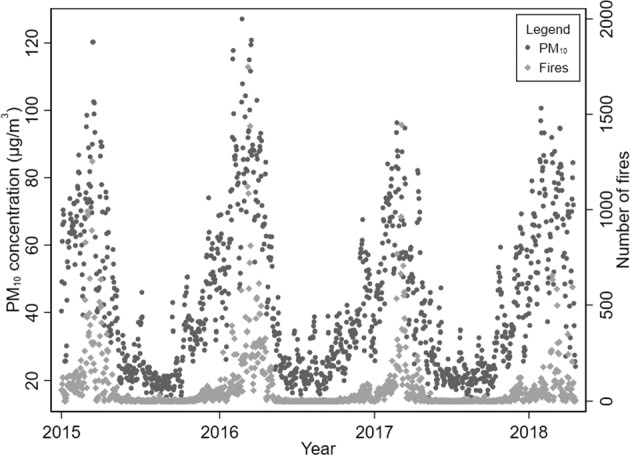
The daily mean PM_10_ concentrations and total number of fires across the study area.

There was a clear seasonal trend in PM_10_ concentrations and the number of daily fires (see Fig. 2). Mean exposure concentrations in each trimester were very similar within each pollutant. Mean entire pregnancy values for PM_10_, O_3_, and NO_2_ were 39.7, 78.1, and 16.5 µg/m^3^, respectively, with an average of 0.15 fires/km^2^ (Table 1). Mean PM_10_ concentrations over the entire pregnancy were strongly correlated with O_3_, moderately so with biomass burning, and weakly with NO_2_ (Table 2).

**Table 2:** Spearman rank correlations of mean pollutant concentrations, number of fires per unit area, and the heat index for the entire pregnancy period.

Pollutant	PM_10_	O_3_	NO_2_	No. of fires	Heat index
PM_10_	1.00				
O_3_	0.74	1.00			
NO_2_	0.37	0.36	1.00		
No. of fires	0.63	0.77	−0.13	1.00	
Heat index	−0.03	−0.11	0.51	−0.26	1.00

**Fig. 3:**
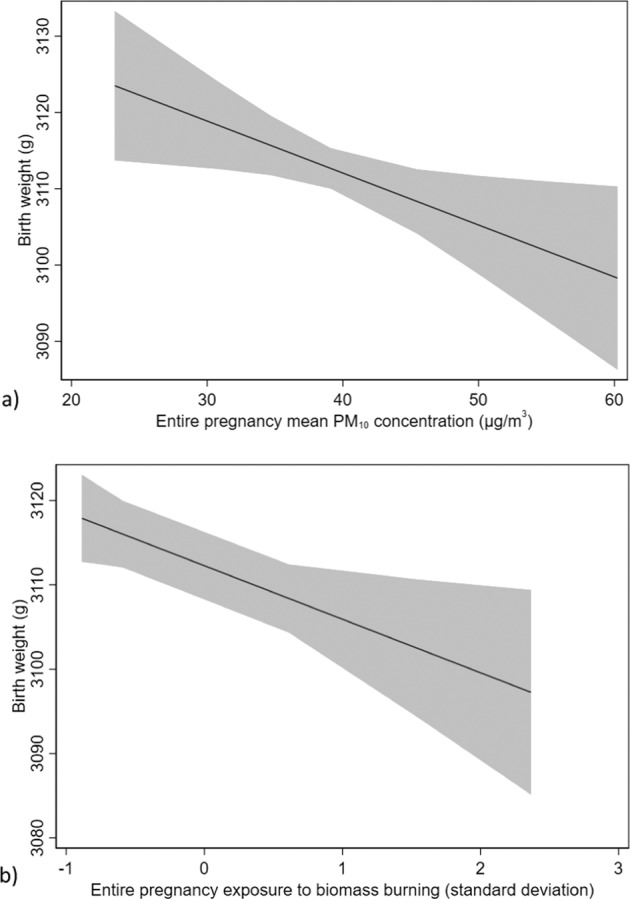
The average birth weight (g) associated with mean whole pregnancy concentrations of **a** PM_10_ and **b** biomass burning across the 1st to 99th percentile of exposure, adjusted for sex, gravidity, maternal age, gestation age, year, province, heat index, and NO_2_ levels.

We identified in unadjusted models a statistically significant decrease in continuous BW per 10 µg/m^3^ increase in PM_10_ concentrations only during trimester one, but in each trimester and the entire pregnancy period for biomass burning. These relationships, however, were attenuated to non-significant levels once potentially confounding variables were included in the model. The additional adjustment for mean NO_2_ levels resulted in a statistically significant decrease in BW associated with both the entire pregnancy mean concentration of PM_10_ and levels of biomass burning (Table 3 and Fig. 3). Additional analyses of effect modification by sex did not appear to show any obvious differential effects for males and females for either PM_10_ or biomass burning exposure (Table S2).

**Table 3:** Change in birth weight (in grams with 95% confidence intervals) associated with a 10 µg/m^3^ increase in PM_10_ and 1 standard deviation increase in biomass burning (bold results are statistically significant).

Exposure	Model 1	Model 2	Model 3
PM_10_			
Trimester 1	**−2.90 (−4.90 to −0.89)**	−0.19 (−2.93 to 2.56)	−0.80 (−4.12 to 2.51)
Trimester 2	−0.86 (−2.47 to 0.75)	2.21 (−0.65 to 5.08)	2.28 (−1.21 to 5.76)
Trimester 3	0.56 (−1.57 to 2.69)	2.33 (−0.54 to 5.19)	0.69 (−2.78 to 4.16)
Entire pregnancy	−3.51 (−7.09 to 0.07)	−2.39 (−6.94 to 2.16)	**−6.81 (−12.52 to −1.10)**
Biomass burning			
Trimester 1	**−9.64 (−12.51 to −6.77)**	−0.54 (−4.62 to 3.55)	−1.39 (−5.83 to 3.06)
Trimester 2	**−7.08 (−9.84 to −4.31)**	0.77 (−3.29 to 4.83)	−1.71 (−6.32 to 2.91)
Trimester 3	**−6.01 (−8.88 to −3.15)**	1.48 (−2.54 to 5.49)	−1.87 (−6.54 to 2.79)
Entire pregnancy	**−12.37 (−15.13 to −9.61)**	−3.12 (−7.42 to 1.17)	**−6.34 (−11.35 to −1.34)**

Model 1 = unadjusted.

Model 2 = adjusted for sex, gravidity, maternal age, gestation age, year, province, heat index.

Model 3 = model 2 + NO_2_.

The adjusted logistic regression analysis suggested a reduced risk of LBW per 10 µg/m^3^ increment of PM_10_ in trimester one. This association maintained after adjustment for NO_2_ levels; mean PM_10_ concentrations in trimester two also reached statistical significance in this model. Conversely, unadjusted models for biomass burning indicated an increased risk of LBW; however, these associations were attenuated once adjusted for potential confounders and also for NO_2_ (Table 4). Additional analyses to assess the presence of interaction of PM_10_ across sex on LBW showed several significant associations of reduced risk in males and females for exposure in different trimesters. For exposure to biomass burning, there was an increased risk only for trimester three exposure in males after adjustment for NO_2_ (Table S3).

**Table 4:** Logistic regression model results (odds ratios with 95% confidence intervals) of PM_10_ (per 10 µg/m^3^) and biomass burning (per 1 standard deviation) exposure with low-birth weight (<2500 g) (bold results are statistically significant).

Exposure	Model 1	Model 2	Model 3
PM_10_			
Trimester 1	0.993 (0.971–1.015)	**0.963 (0.933–0.993)**	**0.953 (0.917–0.991)**
Trimester 2	1.005 (0.988–1.023)	0.968 (0.936–1.001)	**0.942 (0.905–0.981)**
Trimester 3	0.984 (0.961–1.007)	0.973 (0.942–1.006)	0.977 (0.939–1.017)
Entire pregnancy	0.996 (0.957–1.036)	0.987 (0.937–1.040)	0.964 (0.902–1.030)
Biomass burning			
Trimester 1	1.024 (0.993–1.057)	0.981 (0.936–1.028)	0.980 (0.931–1.032)
Trimester 2	**1.034 (1.004–1.065)**	0.993 (0.948–1.040)	0.990 (0.939–1.044)
Trimester 3	**1.033 (1.002–1.065)**	1.014 (0.968–1.061)	1.033 (0.979–1.089)
Entire pregnancy	**1.049 (1.018–1.080)**	1.017 (0.969–1.068)	1.012 (0.956–1.072)

Model 1 = unadjusted.

Model 2 = adjusted for sex, gravidity, maternal age, gestation age, year, province, heat index.

Model 3 = model 2 + NO_2_.

## Discussion

We present findings of the association between pregnant women’s exposure to ambient PM_10_ and biomass burning in several provinces in Thailand and BW as a continuous and dichotomous (<2500 g) outcome. Although there have been a number of previous studies on the impact of PM exposure on BW [3, 4], the current research represents one of the few studies in a low- to middle-income country (LMIC) [37] and, additionally, where women are routinely exposed to biomass burning derived PM. Overall, we identified a weak association between both whole pregnancy PM_10_ and biomass burning exposure and reduced BW as a continuous measure, but only after adjustment for NO_2_. There was little evidence of a heightened risk of these exposures with LBW (limited to trimester three exposure to biomass burning in male births); instead, some associations indicated reduced risks with PM_10_. We discuss our results in relation to previous studies, with special emphasis on those conducted in Asia.

### Continuous BW

In the fully adjusted models examining continuous BW as an outcome, we identified a risk associated with PM_10_ concentrations and biomass burning during the entire pregnancy, but only when adjusting for NO_2_ levels. We did not identify in adjusted models a risk of lower BW in connection with such exposures during individual trimesters. Previous studies of BW in Asia have identified statistical associations between PM exposure and reduced BW, but the timing and risk magnitude have varied. For example, studies have found higher risks with PM exposure in trimesters one (PM_7_ [38]), two (PM_10_ [39]), and three (PM_2.5_ [34]), while Balakrishnan et al. [40] and Xiao et al. [41] found lower BW only with PM_2.5_ concentrations during the entire pregnancy. None of these studies included PM_10_ risk estimates that were adjusted for the presence of other pollutants. Inconsistencies in potential critical windows of exposure also have been observed across the broader (i.e., outside of Asia) literature [4], as well as in the few studies examining exposure to biomass burning or wildfires. Abdo et al. [42] identified lower BW from exposure to wildfire PM_2.5_ in Colorado, USA only during the first trimester, while Holstius et al. [43] found the largest decreases in BW when exposure to wildfires in California, USA occurred during the second trimester; decreases in the first trimester were not statistically significant.

Meta-analyses of single-pollutant models of whole pregnancy exposure to PM_10_ have found reductions in BW ranging from −2.7 g (95% CI: −7.2 to 1.7) [44] to −8.4 g (95% CI: −10.1 to −6.7) [9] per 10 µg/m^3^ increase in PM_10_. As suggested by the positive upper confidence interval in the former, not all research has found BW reductions with coarser PM fractions. In the present study, the effect estimate of the fully adjusted single-pollutant (entire pregnancy) model (−2.4 g per 10 µg/m^3^ [95% CI: −6.9 to 2.2]) was quite close in magnitude to that of Dadvand et al. [44]. More recently, Li et al. [45] did not find any association with BW and PM_10_ exposure in Ningbo, China (but did identify reductions with PM_2.5_). Interestingly, that study identified higher BW with NO_2_ exposure, but did not offer an explanation for this observation. In our study, we found lower BW with PM_10_ and biomass burning occurred only when NO_2_ concentrations were taken into account, which could occur in the presence of a positive association between NO_2_ and BW. A possible explanation of this trend might be that NO_2_ exposures were confounded by the degree of urbanicity: rural areas likely have both lower ambient NO_2_ concentrations [46] and lower BWs [47]. We did not have maternal residential addresses with birth records, so could not control for urban/rural factors in our analysis.

Our results when examining the effect of PM_10_ and biomass burning exposure by sex were mostly consistent with the main analysis, though there were slightly greater decreases associated with biomass burning exposure in females (Table S2). Balakrishnan et al. [40] found a statistically significant decrease in continuous BW only for females (associated with PM_2.5_), which was also identified by Merklinger-Gruchala and Kapiszewska [48] for whole pregnancy PM_10_ exposures. Bell et al. [49] found an adverse association for both male and female infants, though with slightly greater effects in females. In contrast to these findings, O’Donnell and Behie [50] discovered heavier male infants born to mothers exposed to wildfire smoke in Australia. Nevertheless, the point estimates of entire pregnancy exposures for males and females identified in the present study were comparable in magnitude and with most previous findings (i.e., <10 g per 10 µg/m^3^).

### Low birth weight

While BW as a continuous variable was lower on average with entire pregnancy PM_10_ concentrations and biomass burning in the presence of NO_2_, there was a *reduced* risk of LBW with PM_10_ exposure in trimesters one and two; no statistical relationships were apparent among the full pregnancy period for either PM_10_ or biomass burning. Unlike results with the continuous measure, this finding was consistent both with and without NO_2_, although only in the first trimester. ORs reported in meta-analyses of LBW with entire pregnancy PM_10_ per 10 µg/m^3^ are modest, ranging from 1.01 (95% CI: 0.96–1.08) [8] to 1.05 (95% CI: 1.02–1.07) [9], and those with separate analyses for Asian countries [8, 51] show ORs not significantly different from 1.00 for LBW with PM_10_ concentrations. As in the present research, some past studies have found a reduced risk of LBW with PM_10_ exposures, based on land use regression models and ground monitoring data in Brazil [52, 53], trimester one PM_10_ exposures assigned from monitors in China [54], and whole pregnancy PM_2.5_ exposures in Canada based on satellite derived estimates [55]. Habermann and Gouveia [52] showed that in Sao Paulo, Brazil, higher socioeconomic status (SES) was correlated with higher whole pregnancy air pollution exposures; therefore, some of the positive association of SES on BW could have been captured by air pollution indicators in their model. Our study found a protective effect on LBW for exposure in trimester one in both single and multi-pollutant models. As discussed in Fleischer et al. [56], it is possible that the foetus may not survive high exposures during critical periods in gestation. If so, an apparent protective effect in live births of exposure to air pollution might mask any such detrimental effect. There is only weak evidence available to support this hypothesis: Hwang et al. [57] found increased stillbirths in Taiwan with PM_10_ concentrations in gestation months one and two, coinciding with lowered risk estimates observed in trimester one in the present research. Despite these protective associations, our null findings for the entire pregnancy are aligned with findings documented in several cohort studies [4].

In adjusted single-pollutants models, we found a statistically significant protective effect for males with PM_10_ exposure in trimester one and females with trimester three exposures, with no effects for either sex observed for the whole pregnancy. This contrasts with Balakrishnan et al. [40], who found a statistically significant increased risk only for female births and entire pregnancy PM_2.5_. A review based on limited studies found that males might be more susceptible to risks from air pollution due to being less mature at term [58]. Our findings of protective effects with each sex are at odds with this earlier work. A study of biomass burning in Brazil found an ~50% increase in LBW in the highest quartile of PM_2.5_ exposure for trimesters two and three [59]. Our finding of an increased risk in LBW for males and trimester three biomass burning exposure is somewhat consistent with these findings; however, our effect estimate was more modest, and the previous study did not examine sex differences. Further to the discussion above on this topic, these findings of possible effect modification might be attributed to some unmeasured confounder, exposure misclassification, or spurious associations, ultimately suggesting an unclear and/or seemingly weak overall effect of PM_10_ on LBW.

### Strengths and limitations of the study

A major strength of our study was the availability of detailed air pollutant and biomass burning data with individual birth records from a LMIC in a tropical setting. We were able to adjust for some potential maternal confounders, including age and the number of prior pregnancies. Nevertheless, we did not have information on other potentially important confounding variables, such as SES, maternal cigarette smoking, indoor air pollution, and malarial infection. Although the proportion of female smokers is low in Thailand (~2% [60]), the foetus still may have been exposed to second-hand smoke, which would have had a detrimental effect on BW [61]. Likewise, we did not account for indoor air pollution, which has been shown to have adverse effects with birth outcomes [62], nor did we account for different sources of PM. Malarial infections during pregnancy may also influence BW, but there were only about 100 cases of malaria reported during the study period [63], so it is not likely to have had a substantial impact on our results. Other behaviours (e.g., alcohol consumption) that were not accounted for in our analysis and do not differ greatly by season are not likely to have confounded our findings [2]. There was evidence in our dataset that BWs were being rounded to the nearest 10 g and, less commonly, 100 g, which has been previously reported in both LMICs [64] and higher income countries [65] to various degrees. This trend, assuming no bias in the direction of rounding, would introduce imprecision to risk estimates or, more extremely, lead to the inability to detect an effect. Therefore, rounding might have attenuated the estimates in the present study for the continuous birth outcome. In addition, we were not able to exclude multiple births, which, as with rounding, might have attenuated statistical associations. Ultimately, even assuming a significant decreased association between PM and BW, there is still too much uncertainty to quantify health risks later in life based on the magnitude of observed BW reductions (i.e., ~10 g) [6], in part because studies tend to examine such risks on a much larger scale (e.g., per 1 kg increment) [66].

We used ground monitors to assign exposure, which are a somewhat crude indicator of air pollution levels and may not capture important spatial differences in exposure within smaller areas [67]. Further, this source of exposure data may be more problematic when comparing particles and reactive gases, such as NO_2_ and O_3_; gases may exhibit different spatial and temporal distributions [68]. Further, we did not have sufficient PM_2.5_ data to examine associations with BW, which may have produced different findings. Monitors in Thailand, as elsewhere, tend to be situated in more urban or other areas of higher ambient concentrations for regulatory purposes [69]. Thus, exposures assigned to each BW in our study might have been inflated, which would have lessened the magnitude of any resulting air pollution risk estimates (similar to the aforementioned rounding effect). In contrast with the spatially limited PM_10_ exposure data, our exposure metric for biomass burning included fires from across each province; the similarity of results among PM_10_ and biomass burning with continuous BW therefore may underscore the importance of temporal over spatial variability in exposures. Due to the high correlations observed in our study between pregnancy exposure levels of PM_10_ and O_3_, we were unable to distinguish PM effects from O_3_ on BW. These elevated ambient O_3_ concentrations may have occurred downwind from biomass burning [70].

## Conclusion

We present here one of the few studies examining air pollution and BW in a tropical LMIC where biomass burning is widespread and an important source of PM emissions. Our findings suggest a potential association between exposure to ambient PM_10_ concentrations and biomass burning with reduced BW as a continuous measure; however, there was little indication of a clear relationship with LBW (i.e., <2500 g). Although our findings contribute to the relatively small evidence base on the maternal health effects from short- to medium-term ambient air pollution events [18], our study is based on province-level PM_10_ exposures and should be refined in future research to better account for spatial variability and to include exposure to PM_2.5_. While much of the biomass burning occurs in the north of Thailand, PM can travel long distances and has been documented in Bangkok [71]; thus, these potentially harmful exposures are not localised. As populations in LMICs typically are exposed to higher PM levels, the evidence base in these areas in particular should be expanded to help inform policy efforts of air pollution reduction.

## Supplementary information

Supplementary Material
