# Supplementary material for: Exposure to ambient particulate matter and biomass burning during pregnancy: associations with birth weight in Thailand
Source: J Expo Sci Environ Epidemiol. 2021 Feb 18;31(4):672–82. doi: 10.1038/s41370-021-00295-8 (PMC8263346; doi:10.1038/s41370-021-00295-8)
Supplement: Supplementary file 1 — Supplementary Material [file 41370_2021_295_MOESM1_ESM.docx]

**Supplementary Material**

**Table S1.** The coordinates and locations of the Thailand air pollution ground monitors included in the analysis.

| **Region** | **Province** | **Latitude** | **Longitude** |
| --- | --- | --- | --- |
| Central/Eastern | Rayong | 12.97378 | 101.2129 |
| Central/Eastern | Rayong | 12.67155 | 101.2759 |
| Central/Eastern | Chon Buri | 13.05455 | 101.0981 |
| Central/Eastern | Chon Buri | 13.35462 | 100.9792 |
| North | Lampang | 18.27833 | 99.50647 |
| North | Lampang | 18.25082 | 99.76395 |
| North | Lampang | 18.42678 | 99.75763 |
| North | Lampang | 18.28263 | 99.65982 |
| North | Nakhon Sawan | 15.68623 | 100.1106 |
| North | Nan | 18.78888 | 100.7763 |
| North | Phrae | 18.12837 | 100.1624 |
| North | Phayao | 19.16668 | 99.89688 |

**Table S2.** Effect modification by sex for the change in birth weight (in grams with 95% confidence interval) associated with a 10 µg/m^3^ increase in PM_10_ and 1 standard deviation increase in biomass burning (bold results are statistically significant).

| **Exposure** |  | **Model 2** | **Model 3** |  |
| --- | --- | --- | --- | --- |
| PM_10_ |  |  |  |  |
| Trimester 1 |  |  |  |  |
| Male |  | -0.80 (-3.88 to 2.28) | -1.42 (-5.02 to 2.19) |  |
| Female |  | 0.44 (-2.66 to 3.53) | -0.18 (-3.80 to 3.43) |  |
| Trimester 2 |  |  |  |  |
| Male |  | 1.83 (-1.39 to 5.06) | 1.89 (-1.89 to 5.68) |  |
| Female |  | 2.63 (-0.65 to 5.91) | 2.69 (-1.14 to 6.51) |  |
| Trimester 3 |  |  |  |  |
| Male |  | 3.02 (-0.19 to 6.23) | 1.65 (-1.94 to 5.25) |  |
| Female |  | 1.56 (-1.73 to 4.84) | 0.18 (-3.48 to 3.84) |  |
| Entire pregnancy |  |  |  |  |
| Male |  | -2.91 (-8.53 to 2.71) | **-7.96 (-13.23 to -2.68)** |  |
| Female |  | -1.92 (-1.84 to 3.90) | **-6.87 (-12.27 to -1.47)** |  |
| Biomass burning |  |  |  |  |
| Trimester 1 |  |  |  |  |
| Male |  | -1.10 (-5.93 to 3.72) | -1.96 (-7.09 to 3.18) |  |
| Female |  | 0.06 (-4.83 to 4.95) | -0.79 (-5.98 to 4.41) |  |
| Trimester 2 |  |  |  |  |
| Male |  | 1.48 (-3.32 to 6.29) | -1.00 (-6.29 to 4.29) |  |
| Female |  | 0.02 (-4.85 to 4.89) | -2.44 (-7.78 to 2.89) |  |
| Trimester 3 |  |  |  |  |
| Male |  | 4.33 (-0.41 to 9.06) | 1.48 (-3.56 to 6.52) |  |
| Female |  | -1.65 (-6.52 to 3.22) | -4.59 (-9.78 to 0.61) |  |
| Entire pregnancy |  |  |  |  |
| Male |  | -1.47 (-6.47 to 3.53) | **-4.93 (-8.85 to -1.02)** |  |
| Female |  | -4.86 (-9.93 to 0.21) | **-8.37 (-12.38 to -4.35)** |  |
| Model 2 = adjusted for sex, gravidity, maternal age, gestation age, year, province, heat index | | | |  |
| Model 3 = model 2 + NO_2_ | | | | |

**Table S3**. Effect modification by sex (odds ratios with 95% confidence interval) for PM_10_ (per 10 µg/m^3^) and biomass burning (per 1 standard deviation) exposure with low birth weight (<2,500 g) (bold results are statistically significant).

| **Exposure** |  | **Model 2** | **Model 3** |  |
| --- | --- | --- | --- | --- |
| PM_10_ |  |  |  |  |
| Trimester 1 |  |  |  |  |
| Male |  | **0.946 (0.912 to 0.982)** | **0.937 (0.897 to 0.979)** |  |
| Female |  | 0.975 (0.942 to 1.009) | 0.965 (0.926 to 1.006) |  |
| Trimester 2 |  |  |  |  |
| Male |  | 0.971 (0.934 to 1.009) | **0.945 (0.904 to 0.988)** |  |
| Female |  | 0.966 (0.931 to 1.002) | **0.940 (0.900 to 0.981)** |  |
| Trimester 3 |  |  |  |  |
| Male |  | 0.987 (0.950 to 1.025) | 1.006 (0.964 to 1.049) |  |
| Female |  | **0.961 (0.926 to 0.998)** | 0.980 (0.940 to 1.022) |  |
| Entire pregnancy |  |  |  |  |
| Male |  | 0.985 (0.920 to 1.055) | 1.010 (0.946 to 1.078) |  |
| Female |  | 0.989 (0.928 to 1.053) | 1.014 (0.955 to 1.076) |  |
| Biomass burning |  |  |  |  |
| Trimester 1 |  |  |  |  |
| Male |  | 0.942 (0.997 to 1.000) | 0.941 (0.883 to 1.003) |  |
| Female |  | 1.011 (0.959 to 1.065) | 1.009 (0.953 to 1.068) |  |
| Trimester 2 |  |  |  |  |
| Male |  | 0.985 (0.930 to 1.043) | 0.982 (0.923 to 1.046) |  |
| Female |  | 0.999 (0.947 to 1.053) | 0.996 (0.939 to 1.056) |  |
| Trimester 3 |  |  |  |  |
| Male |  | 1.045 (0.990 to 1.104) | **1.072 (1.011 to 1.136)** |  |
| Female |  | 0.985 (0.933 to 1.040) | 1.011 (0.954 to 1.072) |  |
| Entire pregnancy |  |  |  |  |
| Male |  | 1.005 (0.947 to 1.066) | 1.009 (0.962 to 1.059) |  |
| Female |  | 1.027 (0.971 to 1.086) | 1.031 (0.987 to 1.077) |  |
| Model 2 = adjusted for sex, gravidity, maternal age, gestation age, year, province, heat index | | | |  |
| Model 3 = model 2 + NO_2_ | | | | |
